# Validating Force Sensitive Resistor Strip Sensors for Cardiorespiratory Measurement during Sleep: A Preliminary Study

**DOI:** 10.3390/s23083973

**Published:** 2023-04-13

**Authors:** Mostafa Haghi, Akhmadbek Asadov, Andrei Boiko, Juan Antonio Ortega, Natividad Martínez Madrid, Ralf Seepold

**Affiliations:** 1 Ubiquitous Computing Laboratory, Department of Computer Science, HTWG Konstanz, 78462 Konstanz, Germany; akhmadbek.asadov@htwg-konstanz.de (A.A.); andrei.boiko@htwg-konstanz.de (A.B.); ralf.seepold@htwg-konstanz.de (R.S.); 2 Computer Science Department, University of Seville, 41012 Seville, Spain; jortega@us.es; 3 Internet of Things Laboratory, School of Informatics, Reutlingen University, 72762 Reutlingen, Germany; natividad.martinez@reutlingen-university.de

**Keywords:** ballistocardiography, sleep monitoring, wavelet signal processing, heart rate, respiration rate, noninvasive sleep measurement

## Abstract

Sleep disorders can impact daily life, affecting physical, emotional, and cognitive well-being. Due to the time-consuming, highly obtrusive, and expensive nature of using the standard approaches such as polysomnography, it is of great interest to develop a noninvasive and unobtrusive in-home sleep monitoring system that can reliably and accurately measure cardiorespiratory parameters while causing minimal discomfort to the user’s sleep. We developed a low-cost Out of Center Sleep Testing (OCST) system with low complexity to measure cardiorespiratory parameters. We tested and validated two force-sensitive resistor strip sensors under the bed mattress covering the thoracic and abdominal regions. Twenty subjects were recruited, including 12 males and 8 females. The ballistocardiogram signal was processed using the 4th smooth level of the discrete wavelet transform and the 2nd order of the Butterworth bandpass filter to measure the heart rate and respiration rate, respectively. We reached a total error (concerning the reference sensors) of 3.24 beats per minute and 2.32 rates for heart rate and respiration rate, respectively. For males and females, heart rate errors were 3.47 and 2.68, and respiration rate errors were 2.32 and 2.33, respectively. We developed and verified the reliability and applicability of the system. It showed a minor dependency on sleeping positions, one of the major cumbersome sleep measurements. We identified the sensor under the thoracic region as the optimal configuration for cardiorespiratory measurement. Although testing the system with healthy subjects and regular patterns of cardiorespiratory parameters showed promising results, further investigation is required with the bandwidth frequency and validation of the system with larger groups of subjects, including patients.

## 1. Introduction

Sleep is crucial for general health and well-being and its associated problems and diseases can significantly impact daily life, negatively impacting a person’s physical, emotional, and cognitive health through various influencing elements, including hormone control, memory consolidation, and restoration for healing damaged cells. Many mental health conditions, such as sadness, anxiety, and irritability, can be exacerbated by sleep deprivation [1,2,3]. Persistent sleep deprivation has been associated with, for example, a higher risk of obesity, diabetes, high blood pressure, and heart diseases [4].

Circadian rhythm disruption, weakened immunological response, and cardiovascular disease are just a few of the physiological effects of irregular sleep patterns that can substantially affect the body [5,6].

Insomnia [7], sleep apnea [8], restless leg syndrome (RLS) [9], narcolepsy [10], parasomnias [11], and sleep-related movement disorders [12] are some of the most common sleep disorders. Stress, anxiety, depression, loud snoring, daytime sleepiness, disrupted sleep and daytime fatigue, disrupted nighttime, sleepwalking, and sleep talking are some known consequences of such sleep disorders that affect the quality of life and, therefore, require diagnosis or progress evaluation.

The traditional approaches to diagnosing sleep issues involve a combination of/or a clinical assessment [13] and sleep diary, a review of medical history [14], and/or objective measurements of sleep [15,16]. The latter is addressed by methods such as multiple sleep latency test (MSLT) [17], actigraphy [18], and polysomnography (PSG) [19].

PSG is the most accurate and leading sleep study method. It involves monitoring an individual’s brain activity, eye movement, heart rate, and breathing during sleep but not limited to these. It is typically conducted in a sleep lab. However, this approach is obtrusive, time-consuming, and expensive. An effort has been made to develop noninvasive, unobtrusive, and low-cost in-home continuous monitoring systems during the last decades [20,21]. Recent studies have shown that sleep monitoring, including sleep stages, sleep quality assessment, and sleep disorder diagnostics are mainly feasible by measuring cardiorespiratory and body movement, i.e., minimal physiological and non-physiological parameters [22,23,24,25].

Hence, the recent advances in technology have led to the development of new approaches to measure cardiorespiratory parameters during sleep using wearable devices, e.g., pneumobelt sensors [26], smartphone apps [27], video monitoring [28], infrared thermography [29], and ambient sensors [30] to overcome the cumbersome of using PSG. However, interfering with sleep or, to some extent, obtrusiveness, privacy violation and intrusiveness, lack of reliability and accuracy of measurements, complex systems, and positioning uncertainty are some of the reasons transitions in the approaches to further simple, unobtrusive, noninvasive, and reliable measurements [31] are necessary. With advancements in sensor technology from one side and signal processing techniques from the other, methods such as ballistocardiography (BCG) attracted more attention. It is a noninvasive method of measuring the mechanical forces generated by the heart’s contraction during each heartbeat [32,33].

BCG signals are measured using sensors placed on or under a subject’s body, typically on/under a bed or a chair. Several different types of sensors can be used for BCG measurements during sleep, and the number and types of sensors continue to evolve as new technology emerges. Pressure/force-based sensors such as force-sensitive resistors (FSR) [34,35], load cells [36], inertial sensors such as accelerometers, gyroscopes, and magnetometers [37,38], capacitive micromachined ultrasound transducers (CMUTs) [39], piezoelectric sensors [40], and strain gauges [41] have been tested and used in cardiorespiratory measurement during sleep.

In addition, fiber-optic sensors have been used in BCG measurements as an alternative to traditional mechanical sensors [42]. There are several different types of fiber-optic sensors, including fiber Bragg Grating (FBG) [43], Fabry–Perot interferometer [44], and microbend [45]. These sensors use the structure inscribed in a fiber-optic cable to measure strain and displacement. Fiber-optic sensors offer several advantages over traditional mechanical sensors, including high sensitivity, noninvasiveness, and immunity to electromagnetic interference [46]. However, they are expensive and require specialized equipment to operate, which can limit their widespread use in sleep monitoring [47].

As an example of noninvasive and unobtrusive measuring systems, the Medical Automation Research Center at the University of Virginia developed a noninvasive analysis of a physiological signals system to measure physiological and environmental parameters [48]. In this system, two resilient force-coupling pads were placed beneath a typical hospital bed sheet to detect the tiny forces generated during heart contraction and relaxation. Additionally, the system was able to recognize changes in posture, breathing exertion, and body movements. The system was examined and validated on 40 healthy participants during overnight research. Compared to the reference ECG, pulse oximetry, and respiratory inductance PSG, the system produced good output [49].

More in the direction of diagnosis and progress evaluation, there are a few methods specifically for inconspicuous apnea detection. For unconstrained apnea and arousal detection, for instance, Mack et al. recommended employing the NAPSTM system; the proposed approach was validated using data from forty participants [50]. In a dataset with 32 subjects, Hwang et al. suggested using a polyvinylidene fluoride film sensor put on top of the mattress to identify apneic occurrences [51]. For 45 participants, Beattie et al. described the use of load cells situated beneath the bed’s supports to identify apneic episodes, with the apneic detection being performed manually by a professional [52]. In order to identify apneic and limb movement events for patients. Waltisberg et al. devised a sensor with integrated strain gauges implanted beneath the bed mattress [53].

In order to evaluate the potential of a single mat with an embedded microbend fiber-optic sensor for nonintrusive monitoring of vital signs and the absence of breathing, a pilot feasibility study was carried out in a clinical trial setup. The microbend fiber-optic sensor pad was positioned beneath the patient’s chest and stomach. Despite the considerable body movements during apnea occurrences, the system established a good agreement with the reference device. Moreover, the mean absolute error of the mean respiratory and cardiac rates was quite low [54]. A noninvasive and unobtrusive system using FSR sensors installed under the mattress on the bed frame was proposed to measure heart and respiratory rates. The authors reported an average error of 4 BPM in contrast with the reference device [55].

Another low-cost yet noninvasive and contactless system using off-the-shelf sensors, i.e., load cell, was proposed in [56]. The sensors were installed on a typical hospital bed to measure the longitudinal BCG. It aimed to evaluate its utility for monitoring heart and respiration rates. An unsupervised machine learning algorithm was deployed to evaluate its performance to an electrocardiogram (ECG) signal that serves as a reference. The system was tested with seven subjects in four different sleeping positions and delivered an overall detection rate of 83.9%.

The authors in [57] have developed an unobtrusive and noninvasive monitoring system for sleep-related breathing disorders (SRBDs) and in particular, obstructive sleep apnea (OPA). The system is Arduino-based, running FSR sensors embedded in the pillowcase and the bedsheet. The system is further enriched with a triple-axis accelerometer sensor (ADXL345) and a microphone. The platform analyzes the body position, respiratory rate, snoring, sleep efficiency, and sleep apnea. The system also is an effort in order to train the subject to sleep in an appropriate position to avoid snoring. The authors reported the high agreement of the system in comparison with the PSG as the gold standard.

The authors in [58], used flexible piezoresistive architectures and machine learning algorithms to develop an integrated sleep monitoring system. This low-cost and privacy-protecting system includes a flexible pressure-sensing pad, in the form of a piezoresistive array for capturing the pressure distribution during sleep, and the readout circuit. The authors reported a high sleeping posture classification accuracy of 98.1% and RR estimation accuracy of 97.5%, indicating a strong potential in practical utilization.

Gaiduk et al. in [59], developed an unobtrusive and low-cost sleep analysis system based on a hardware sensor net. It is a grid of 24 pressure sensors, supporting sleep phase recognition, respiration, and body movement. The hardware configuration of the system includes a series of pressure sensor nodes forming a mesh architecture connected to a microcontroller via a system-wide bus with address arbitration. All nodes are connected. The embedded system enables network configuration, storing and pre-processing of the data, external data access, and visualization. The authors reported validating the system with healthy young subjects. The results obtained have indicated the potential to detect breathing rate and body movement.

One of the main challenges with using BCG for sleep measurement is related to the morphology of the signal. The BCG signal is characterized by a complex waveform that is influenced by various factors, including body movements, the position of the subject on the bed, and the location of sensor deployment. Nevertheless, the choice of the sensor itself depends on several factors, including the research question, application, the desired accuracy and resolution of the data, and the available resources and infrastructure. Moreover, the signal contamination by sources of noise, such as vibrations from external sources or movements of the bed, the non-stationarity of the signal, signal variability, and lack of standardized analysis methods, can make it difficult to develop a universal algorithm for sleep measurement using BCG. All these factors influence the quality of the signal processing and analysis algorithms [60].

In this work, we develop and propose an optimal, reliable, unobtrusive, and noninvasive cardiorespiratory measuring, continuous in-home system with low hardware setup complexity and user-friendliness to estimate heart and respiratory rates. This is accomplished by testing and validating a strip standard FSR sensor. It further contributes to reducing the dependency of accuracy on different sleeping positions. Furthermore, we improve the reliability of the measurements by implementing two algorithms for heart rate and respiration measurements using discrete wavelet transformation (DWT) and 2nd-order Butterworth bandpass filter, respectively. The algorithms are light, efficient, near-real-time operating, and do not need a complex embedded system.

The rest of this paper is organized as follows: in Section 2 the mechanism of the system development, signal processing techniques, and data acquisition are described. In Section 3 the results are presented. The paper is followed with the Discussion including the interpretation of the data, the applications and extendibility, and the restriction of the work. It is ended with the Conclusions.

## 2. Materials and Methods

### 2.1. System Setup and Configuration

The system consists of (i) two FSR 408 strip sensors, (ii) two amplification boards, (iii) one analog-to-digital converter (ADC) board as well as ADC to an inter-integrated circuit (IIC) interfacing converter, and (iv) an embedded system. The edging measuring sensors, pre-processing, transmission and interfacing, and data processing occurs in steps one to four, respectively.

#### 2.1.1. Sensor

FSR 408 strip is a single-zone robust polymer thick film (PTF) sensor with 622.3 mm in length that exhibits a decrease in resistance with an actuation force of as low as 0.2 N with a sensitivity range of up to 20 N.

#### 2.1.2. Pre-Processing, Communication, and Interfacing

The sensor is directly plugged into the pre-processing board to amplify and filter the signal and gain. The board is designed based on the single supply chain operational amplifier TLC271ACD (Texas Instruments, Dallas, TX, USA), which combines a wide range of input offset voltage grades with low offset voltage drift and high input. The lower and higher boundary of the low- and high-pass filters are set to 0.15 and 15 Hz, respectively, which correspond to the cardiorespiratory signals to detect. We also consider that the mattress, the subject, and the sensors act as low-pass filters. The signal gain is set to 90 to overcome the distortion and low amplitude of the signal (<0.5 mV). As the embedded system does not support the analog signal, we utilize ADS1015 (Adafruit, New York, NY, USA), an ADC, and a switching communication protocol from ADC to IIC. ADS1015 is high precision, four single-ended input channels supporting 12-bit precision at 3300 Hz over IIC.

#### 2.1.3. Embedded System

A Raspberry Pi 4B (Raspberry Pi foundation, Cambridge, UK) with 4 GB RAM and 32 GB external memory is used to collect, store, and process the data.

### 2.2. Experiment Setup and Study Design

#### 2.2.1. Bed and Mattress

We used a regular single bed (Askvoll), bed net/slatted (Lönset), and mattress with dimensions of 90×200 from IKEA (IKEA, Delft, The Netherlands). We have not considered any particular requirements or customized specialties. All materials were wooden and widely accessible by regular users.

#### 2.2.2. Sensors Deployment and Distributions

**Figure 1:**
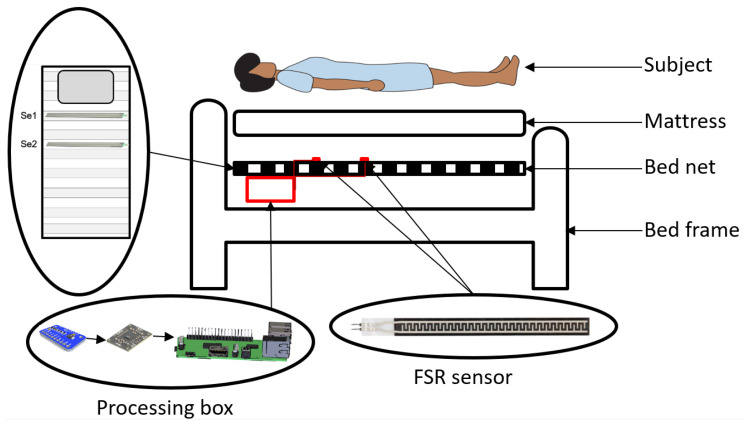
The approach offers a noninvasive, nonintrusive, and unobtrusive measurement of cardiorespiratory parameters. The sensors and system setup are located under the mattress and attached to the bed net without inconveniencing the user.

We utilized two FSR strip sensors of Se1 and Se2 on the bed net and under the mattress to cover subjects’ thoracic and abdominal regions, respectively, measuring the cardiorespiratory parameters. The sensors are deployed horizontally, covering approximately 62 cm of the bed. Sensors are connected to the pre-processing boards, ADC over the IIC board, and eventually linked to Raspberry Pi 4B via wire (see Figure 1).

#### 2.2.3. Subject Description

We recruited 20 subjects, 12 males and 8 females, with an average age, height, and weight of 31 ± 8 years, 173 ± 8 cm, and 71 ± 7 kg, respectively. All subjects were healthy and informed of the consent form. The subjects did not acknowledge any known cardiovascular, pulmonary, or other diseases, nor were they under any treatment or medication.

#### 2.2.4. Data Acquisition

**Figure 2:**
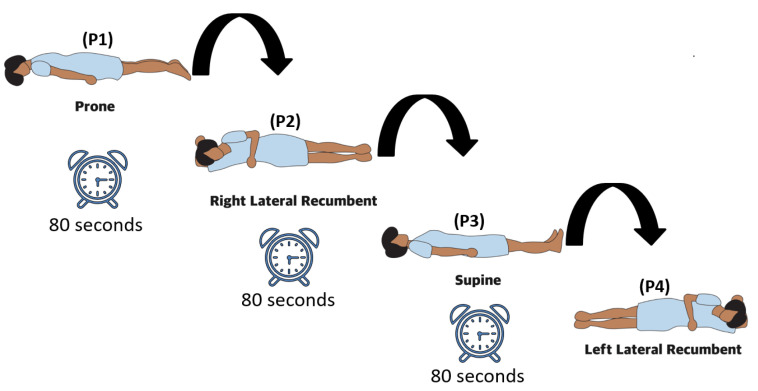
We have performed the experiment in all four regular sleeping positions. The subject switched position after every 80 s.

We used SOMNO HD eco PSG (SOMNO medic GmbH, Randersacker, Germany) to record the reference data. We recorded the respiratory (thorax and abdomen) and ECG signals at 32 Hz and 256 Hz, respectively. The belt was tightened to a comfortable level, and ECG electrodes were disposable. Data from the FSR strip sensors were captured at a sampling rate of 150 Hz. The subjects were instructed to lie down on the bed in four different positions: prone (P1), right lateral (P2), supine (P3), and left lateral (P4). The experiment started with the individuals prone and ended with the left lateral position in a counterclockwise rotation of the subjects. The data measurement lasted 80 s in each position. We gave subjects five minutes to relax before collecting data. During the data collection, the subjects were instructed to behave normally with the least amount of movement. They were nevertheless advised that in the event of an inconvenience, the experiment would be stopped (see Figure 2).

### 2.3. Data Processing and Analysis

We performed the data synchronization between the FSR strip sensors data and the reference signals after removing the first and last ten seconds of the FSR strip sensors data in each position and the offset. We derived the BCG and breathing signals from FSR strip sensors. We extracted the BCG and respiratory signals using the Chebyshev type I bandpass filter with the filters’ lower and higher cutoff frequencies (2.5–5 Hz, 0.5 dB) and (0.01–0.4 Hz, 0.5 dB), respectively. The data recording and processing were performed in a chunk of 20 s using a sliding time window size of 3000 samples.

**Figure 3:**
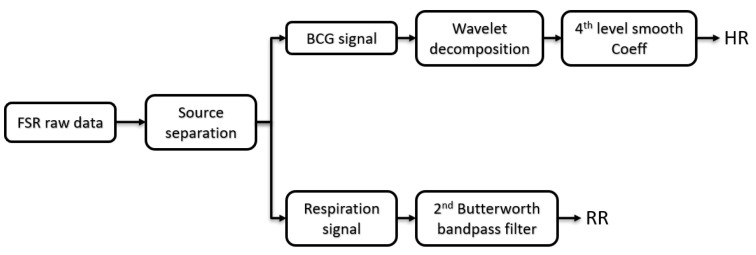
The signal processing was performed in two pipelines using discrete wavelet transform and Butterworth bandpass filter.

To calculate the heart rate (HR), the multi-resolution analysis with the maximal overlap discrete wavelet transform (MODWT) was performed. The BCG signal was converted into an approximation and detailed signal by passing it through low- and high-pass filters. The coefficients of the filter were not subsampled. After performing several trials earlier than the actual data acquisition and observing the agreement between the periodicity of the maxima and the cardiac cycles, the 4th smooth level (lev = 4) of wavelet biorthogonal 3.9 (bior3.9) basis function was chosen for the decomposition process to estimate HR. Biorthogonal wavelet filters generate one scaling function and wavelet for decomposition, and another pair for reconstruction. MODWTMRA is zero-phase filtering of the signal. Features will be time-aligned. LoD, HiD, LoR, and HiR are the four lowpass and highpass, decomposition (LoD and HiD) and reconstruction (LoR and HiR) filters associated with the biorthogonal wavelet, respectively (see Figure 3).

We used a Butterworth 2nd order as the bandpass filter in the frequency range of 0.15 to 0.4 Hz to estimate respiration rate (RR). This step was followed with a peak detector to recognize the respiratory peaks.

We assessed the outcomes using mean absolute error (MAE) and Bland–Altman limit of agreement (LoA) for processing and analysis.

## 3. Results

**Figure 4:**
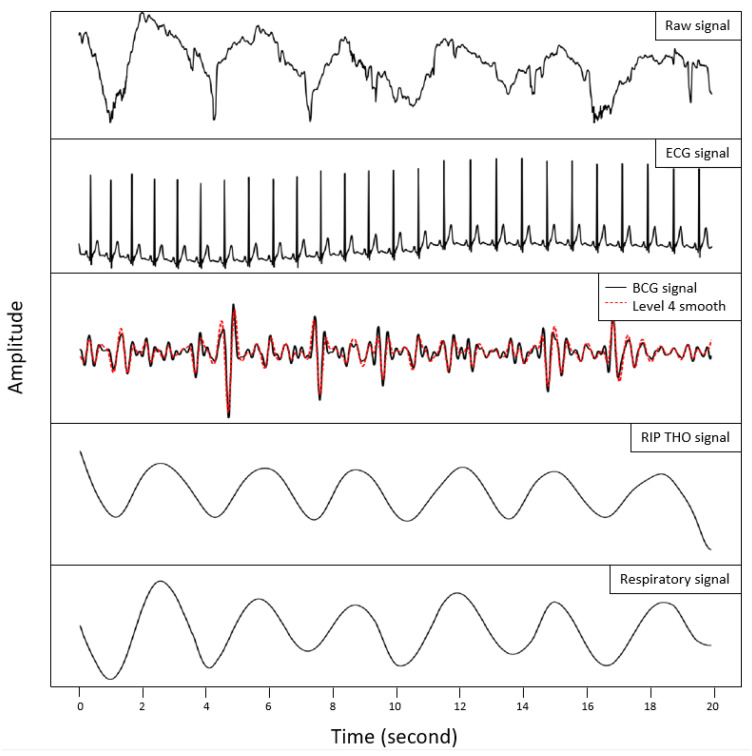
The label description of the figure from top to bottom are as follows: the acquired signal from the strip sensor under the thoracic region (Raw signal), the reference ECG signal as the gold standard (ECG signal), BCG signal and the processed signal acquired from the strip sensor for HR estimation (BCG signal and 4th smooth level), the reference respiration signal from the thoracic region as the gold standard (RIP THO signal), and the respiration signal from the strip sensor under the thoracic region (Respiration signal): 20 s of the 9th subject’s signals in P4 position. The sensitivity of the FSR strip enabled us to detect the smallest movements caused by cardiac and breathing activity.

We processed the data after cutting the first and last ten seconds of signal in each position and, thus, prepared 60 s in each position and 240 s for each subject in all four positions. In total, 4800 s of data were divided into 240 signal segments for HR and RR estimation (see Figure 4).

In RR estimation of all subjects in all positions, Se1 and Se2 delivered the average MAE of 2.32 and 2.83, respectively. Dividing the results according to gender showed that the average MAE of RR estimation for Se1 and Se2 was 2.33 and 2.77 for females, and 2.32 and 2.86 for males, respectively.

The RR estimation from the thoracic region (Se1) yielded the same results for both genders (MAE = 2.32). The abdominal region (Se2) also yielded similar outputs for both genders, slightly better for females (MAE < 0.09). In total, estimating RR from the thoracic region delivered better results than abdominal regions in males, females, and all subjects.

Comparing the average MAE results of Se1 and Se2 in individual positions for all subjects indicated similar results. Considering both genders, Se1 in individual positions suppressed Se2 with a lower error, even with a small difference. Further, exploring Se1 as the superior sensor showed that Se1 delivers the smallest and largest error in P1 (2.09) and P3 (2.57), respectively. However, P2 with an error of 2.13 and P4 with 2.49 are in the same range, i.e., improving the accuracy of the measurements has reduced the sleeping position dependency. This pattern was in line for males where Se1 in individual positions delivered lower error than Se2. It is repeated for females with an exception in P4. Although the outputs of Se1 in P1 to P3 overcome Se2, the opposite results were observed in P4. This could be neglected due to the very small difference (MAE = 0.14). The significant comparison of Se1 and Se2 takes place for females in P1, delivering MAE of 1.85 and 3.17, respectively.

The most minor and most significant deviations between sensors in the same position were observed in P4 (0.03) and P1 (0.86). For females, P4 (0.14) and P1 (1.32) and for males, P4 (0.09) and P2 (0.82) delivered the smallest and largest deviations, respectively (see Table 1).

From the LoA point of view, Se2 stud better than Se1 with a total LoA in the range of [−6.19, 5.98] for all subjects, [−6.20, 6.25] for males, and [−6.16, 5.29] for females.

As for HR estimation of all subjects and positions, Se1 and Se2 delivered average MAE of 3.24 and 3.67, respectively. These results for females were 2.68 and 3.94, and for males, they were 3.47 and 3.55, respectively. In total, measuring HR from the thoracic region in males, females, and all subjects suppressed the abdominal region. For individual positions of all subjects, Se1 yielded superior results to Se2. Further, exploring Se1 as the efficient sensor indicated that the smallest and largest errors are yielded by P3 (2.85) and P1 (3.47), respectively. However, the difference between the error of these two positions is as small as 0.62, i.e., reducing the output dependency on the sleeping positions. For females, Se1 in all four positions gave better results than Se2. P2 with 2.12 and P1 with 3.20 carry the smallest and largest errors, respectively. For the males, Se1 and Se2 showed similar performance with the smallest and greatest error in P3 (2.91) and P2 (4.10) (see Table 2).

Overall, in all four positions, Se1 in the thoracic region performed better, and we reached the error of 2.32 and 3.24 for RR and HR estimation, respectively. Overall, the system showed similar performance and reliability of RR estimation for both males and females (unless P1 for females); but females performed significantly better in HR estimation than males. The females’ RR estimation in P1 was significantly better than males, which can be due to the physical differences in the chest region. As for RR estimation, Se1 performed better than Se2 with minimum dependency on sleeping positions and gender. Regarding HR estimation, for females, Se1 delivered better results in all positions. The error in P2, P3, and P4 (Se1: 2.12, 2.71, and 2.68 compared to Se2: 4.01, 4.45, and 3.85) are significantly different from P1 (Se1: 3.20 compared to Se2: 3.46). However, it could be concluded that Se1 suppressed Se2 in both RR and HR estimation for both genders, with small differences.

**Figure 5:**
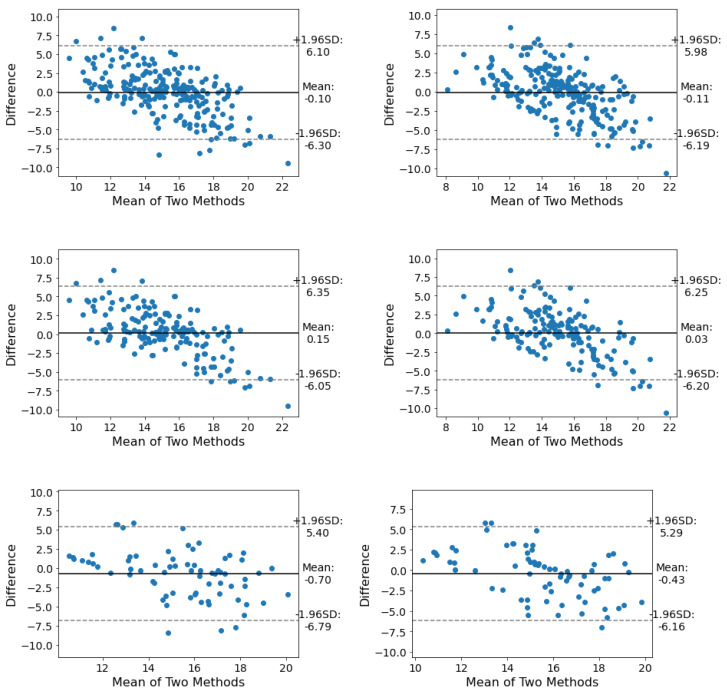
The LoA between the respiratory estimation of Se1 (**left**) and Se2 (**right**) under the thoracic and abdominal regions. From top to bottom: LoA of all subjects, males, and females. In total Se2 showed a better agreement with the reference data.

**Figure 6:**
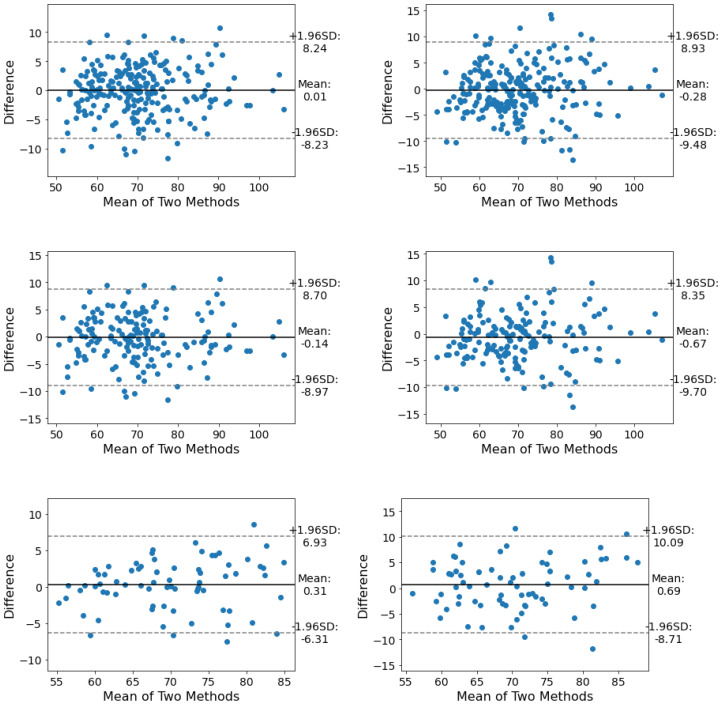
The LoA between the heart rate estimation of Se1 (**left**) and Se2 (**right**) under the thoracic and abdominal regions. From top to bottom: LoA of all subjects, males, and females. For females, Se1 showed significantly better agreement with the reference data.

From the LoA point of view, Se1 has a better LoA than Se2 in all three categories. The overall LoA of Se1 in all positions is in the range of [−8.23, 8.24], [−6.31, 6.93] for females and [−8.90, 8.70] for males (see Figure 5 and Figure 6).

## 4. Discussion

### 4.1. Challenges and Drawbacks

Estimating reliable HR and RR during sleep from the BCG technique using FSR sensors can be challenging for several reasons, such as the signal morphology, signal-to-noise ratio, and sensitivity to changes in pressure that can be affected by changes in body position, body weight, and other factors. One of the known challenges is to design a system with features and specifications such as certainty and reliability of measurements in different sleeping positions, and yet has a simple, optimized, low-cost, and user-friendliness system. This is correlated to the coverage region of the bed and the position of the sensor(s), as well as the sensitivity of the sensor(s). This was addressed in some works using distributed FSR sensors, and fiber-optic solutions [43,57,59,61,62]. However, the first one suffers from a distributed approach and thus lacks accuracy, and dependency on sleeping positions, and the latter, although the reliability of measurement has been improved, the complexity and expense of the system have been increased.

### 4.2. Improvements, Applications, and Opportunities

We tested and validated FSR strip sensors to address the reliability and accuracy of the system, certainty, and reducing the dependency on sleeping positions by sensor sensitivity, seamless signal processing, and covering the larger region of the bed, respectively. Using a minimum number of simple sensors consequently reduced the system’s complexity and the total development cost. We tested the system’s performance with 20 subjects of both genders to perform an in-deep evaluation due to various physical features of males and females influencing the morphology of the signal.

We could noticeably reduce the dependency of the measurements on the sleeping positions in RR and HR estimation of both genders. We reached the error difference of RR estimation (dependency on sleeping positions) of 0.49 and 0.89 for males and females, respectively, and HR estimation of 1.19 and 1.08 for males and females, respectively.

This improvement might be of significance in both clinical trials and OCTS systems where the monitoring subjects are usually patients [18]. They often suffer from a sleep disorder and consequently require an unobtrusive and noninvasive measurement that does not interfere with their sleep nor impose any additional activities, while it is affordable, reliable, and could be referred by clinicians to motivate both parties using it. In addition, such a system with minimal dependency on the sleeping positions in actual life, where there is minimum control on the subjects, can bring additional benefits in terms of acquiring more extended periods of valid signal because the subject does not need (or at least is less required) to follow particular instructions. Moreover, as the overall performance of the system has been improved rather than position-related enhancement, with further tests and validation, we expect re-calibration and personalization are less often needed. Consequently, the low effort and cost of maintenance is achieved.

With the initial tests performed and the validation, the system has shown its potential in sleep stage identification as well as stress monitoring [24,63,64]. These roles can be considered through the accurate measurement of movements and cardiorespiratory as well as heart rate variability implementation. Therefore, this enables the system applications to be extended not only as a diagnostic but well-being monitor—a complementary or substitution to wearable devices which often are considered obtrusive to some extent [65].

The system deployment can depend on the purpose and gender according to the application. Using the system with the optimal configuration (only one sensor) for RR estimation requires Se1 deployment disregarding gender. We expect that in such an application, Se1 delivers a less average MAE (0.5) than Se2. Regarding the HR estimation, depending on the subject’s gender, this needs to be tuned. If females use the system, Se1 should be deployed; and if males use the system, either Se1 or Se2 can be chosen, with a priority to Se1. Care must be taken in particular applications where the system is supposed to be used, for example, only in one position in HR estimation for males.

In general, using Se1 for measuring both cardiorespiratory parameters in both genders yields optimal performance. The body surface and shape of subjects can be impactful in HR estimation, as we observed such a factor reflected the HR estimation of females in Se1 and Se2. However, we could not formulate this point. We found negative (−0.72) and positive (0.82) correlations between the females’ weights and heart rate and respiration rate measured from Se1, respectively. We could not find such a correlation in males.

FSR sensors can carry a sort of error even though tested under the same conditions which can affect the stability and reproducibility of the signal and data. However, we have tried to exclude the effect of external factors by maintaining similar experimental conditions for all subjects. This includes (i) air conditions such as temperature, humidity, and pressure, by continuously measuring and tracking these parameters, (ii) performing the experiments during a fixed time of the day with some tolerance, (iii) leaving the bed unoccupied for a duration of 15 min to recover the potential drift of the sensors to initial states from the previous experiments (influenced by weight, force, and surface of subjects), and (iv) maintaining the environment isolated from sound pollutants. We did not observe any particular divergence, trend, sensitivity loss, or performance drop during the experiment. This could be confirmed by the comparison of the subjects’ sequence experiments over time with MAE. Looking at the weight factor, we could not find any relation between the MAE before or after a subject with significantly different weight than others. This is the same with the genders. We performed the experiments in a completely random gender sequence and the results disregarded the sequence of the experiment. Considering the performance and output of the sensors, the stability of the sensors under the discussed conditions is trustworthy.

Developing a sleep monitoring system is composed of several aspects, of which the reliability of the system, unobtrusiveness, nonintrusiveness, complexity, and expense are given priority. However, depending on the application of the system (diagnostic, well-being, and general health monitoring), mode of usability (in-home monitoring, clinical trials), target group (patients, healthy subjects, elderly, children), element of measurement (sleep staging, sleep-related disorders) and technology use, one or the other gain greater weight. Thus, the previous works depending on the stated criteria have addressed various aspects varies from one to another, which makes the comparison more qualitative rather than quantitative [66].

Reliability of the system: includes accuracy of the measurement (less error compared with the reference data), stability of the system (less failure prone, drift, hysteresis, etc.), and reproducibility of the signal and data. We aimed to validate the cardiorespiratory measurements that play important roles in sleep staging, and sleep-related diseases and abnormalities detection in well-being, general health monitoring, and diagnostics. We attempted to enhance this factor by reducing the dependency on sleeping positions by improving the sensitivity of the sensors. Compared to the works in [34,35,57,58,59] which are using similar technology, the performance of the system in terms of accuracy of the cardiorespiratory parameters has been improved. On the other hand, the fiber-optic-based system in [42,67], in general, delivers greater performance disregarding the complexity and cost of the system.

Unobtrusiveness: does not interfere with the regular sleeping positions nor impose additional activities. Not considering the wearable devices and camera-based vital signs monitoring during sleep, this work and comparable studies such as those in [34,35,42,57,58,59,67] are using the similar method of deployment for the sensors, which are under the mattress and on the bed slatter. However, the work resolves the issue, to some extent, of unobtrusive deployment of FSR sensors in bed and pillow cases [68].

Nonintrusiveness: complies with the privacy of the user and data. Despite of the camera-based approaches presented in [69,70,71], and similar to [34,35,57,58,59] our work preserves the privacy of the subject.

Complexity of the system, maintenance, and expense: includes the software and hardware implementation mechanism which deals with several sensors, construction of the sensors’ net, readout, communication, deployment, and operation. It will impact the user-friendliness of the system, instructions for use, and cost, but not limited to these. The expense of the system is a consequence of implementation and configuration, material and sensors, processing box, embedded system, and the need for calibration and re-calibration at the maintenance stage. Among other things, this is the highlight of our system which, compared to the work in [42,44,55,62], reduces the complexity of sensor net, readout, processing time, deployment, and communication and, unlike the fiber-based sensors, is affordable and does not need complex instructions for use. In addition, due to the simplicity of the system, if required a sensor could be substituted with another by the user.

### 4.3. Limitations

However, it should be noted that we measured the healthy subjects with regular cardiorespiratory parameters. Patients with irregular patterns might demand altered bandwidth frequency. In addition, even though we covered a wide range of HR from 48 to 108 and RR from 8 to 23, the system needs to be evaluated with subjects different than to range. In-depth diaphragm or thoracic breathing could be an influencing factor in the final system evaluation that requires further investigation.

## 5. Conclusions

We designed, developed, and tested a noninvasive, low-cost, simplified, and user-friendly in-home continuous cardiorespiratory system using FSR strip sensors. This preliminary study indicated that increasing the sensitivity of traditional mechanical sensors followed by signal processing can contribute to detecting slight cardiac and respiration activity and, therefore, estimating the heart rate and respiration rate. Using the FSR strip sensors, we could improve the reliability and accuracy of the system to as small as errors of 2.32 and 3.24 for respiration and heart rate, respectively (reflected in the results of sensors delivering less error). In the RR estimation of all subjects in all positions, Se1 and Se2 delivered an average MAE of 2.32 and 2.83, respectively. The average MAE of RR estimation for Se1 and Se2 was 2.33 and 2.77 for females, and 2.32 and 2.86 for males, respectively. In HR estimation of all subjects and positions, Se1 and Se2 delivered an average MAE of 3.24 and 3.67, respectively. These results for females were 2.68 and 3.94, and for males, they were 3.47 and 3.55, respectively. In addition, we could decrease the system’s dependency on the sleeping positions for heart rate and respiration rate to as small as 0.62 and 0.48, respectively. Our study showed that designing an optimized system with minimal deployed sensors is feasible for measuring both males’ and females’ heart and respiration rates from the thoracic region. We observed fewer errors during the heart rate measurement for females than males. In addition, we could find the correlation between the weight and cardiorespiratory results obtained from the thoracic region in females, which is reflected in the results, and justified by the body surface and physical differences in the chest region.

## Figures and Tables

**Table 1:** The respiration estimation shows the agreement on carrying less error by Se1 under the thoracic region compared to Se2 under the abdominal region for both genders and in all positions.

	Male		Female		All Subjects	
Position	*Se*1	*Se*2	*Se*1	*Se*2	*Se*1	*Se*2
*P*1	2.19	2.86	1.85	3.17	2.09	2.95
*P*2	2.04	2.86	2.34	2.52	2.13	2.76
*P*3	2.50	3.11	2.74	3.11	2.57	3.11
*P*4	2.53	2.62	2.41	2.27	2.49	2.52
Average	2.32	2.86	2.33	2.77	2.32	2.83

**Table 2:** The heart rate estimation of Se1 and Se2 between males and females are significantly different. This might be due to the physical difference in the thoracic region.

	Male		Female		All Subjects	
Position	Se1	Se2	Se1	Se2	Se1	Se2
P1	3.58	3.58	3.20	3.46	3.47	3.55
P2	4.10	4.04	2.12	4.01	3.51	4.03
P3	2.91	3.57	2.71	4.45	2.85	3.83
P4	3.31	3.02	2.68	3.85	3.12	3.27
Average	3.47	3.55	2.68	3.94	3.24	3.67

## Data Availability

The data presented in this study are available on request from the corresponding author.
